# CT based three dimensional dose-volume evaluations for high-dose rate intracavitary brachytherapy for cervical cancer

**DOI:** 10.1186/1471-2407-14-447

**Published:** 2014-06-17

**Authors:** Naoya Murakami, Takahiro Kasamatsu, Akihisa Wakita, Satoshi Nakamura, Hiroyoki Okamoto, Koji Inaba, Madoka Morota, Yoshinori Ito, Minako Sumi, Jun Itami

**Affiliations:** 1 Department of Radiation Oncology, National Cancer Center Hospital, 5-1-1, Tsukiji Chuo-ku, Tokyo 104-0045, Japan; 2 Department of Gynecologic Oncology, National Cancer Center Hospital, 5-1-1, Tsukiji Chuo-ku, Tokyo 104-0045, Japan

**Keywords:** Brachytherapy, Image-based gynecological brachytherapy, Cervical cancer, IGBT, CT-based gynecological brachytherapy

## Abstract

**Background:**

In this study, high risk clinical target volumes (HR-CTVs) according to GEC-ESTRO guideline were contoured retrospectively based on CT images taken at the time of high-dose rate intracavitary brachytherapy (HDR-ICBT) and correlation between clinical outcome and dose of HR-CTV were analyzed.

**Methods:**

Our study population consists of 51 patients with cervical cancer (Stages IB-IVA) treated with 50 Gy external beam radiotherapy (EBRT) using central shield combined with 2–5 times of 6 Gy HDR-ICBT with or without weekly cisplatin. Dose calculation was based on Manchester system and prescribed dose of 6 Gy were delivered for point A. CT images taken at the time of each HDR-ICBT were reviewed and HR-CTVs were contoured. Doses were converted to the equivalent dose in 2 Gy (EQD_2_) by applying the linear quadratic model (α/β = 10 Gy).

**Results:**

Three-year overall survival, Progression-free survival, and local control rate was 82.4%, 85.3% and 91.7%, respectively. Median cumulative dose of HR-CTV D_90_ was 65.0 Gy (52.7-101.7 Gy). Median length from tandem to the most lateral edge of HR-CTV at the first ICBT was 29.2 mm (range, 18.0-51.9 mm). On univariate analysis, both LCR and PFS was significantly favorable in those patients D_90_ for HR-CTV was 60 Gy or greater (p = 0.001 and 0.03, respectively). PFS was significantly favorable in those patients maximum length from tandem to edge of HR-CTV at first ICBT was shorter than 3.5 cm (p = 0.042).

**Conclusion:**

Volume-dose showed a relationship to the clinical outcome in CT based brachytherapy for cervical carcinoma.

## Background

Standard therapy for patients with locally advanced cervical cancer is combination of external beam radiotherapy (EBRT) and brachytherapy with concurrent chemotherapy [1-5]. Intracavitary brachytherapy employing intrauterine (tandem) and vaginal (ovoid) sources based on Manchester principles, has been the standard for many decades [6,7]. Manchester system is point-based (i.e. two-dimensional) and uses orthogonal x-ray images for calculation and prescription of treatment doses. This concept neglects each tumor size or shape because prescribed dose is delivered to a fixed reference points. Therefore while excellent long-term tumor control rates can be obtained for patients with small tumors, for larger tumors relapse rate are high [8,9]. Over the decades, GEC-ESTRO [10,11] and ABS [12] proposed the concept of 3D image-based brachytherapy (IGBT) for the cervical cancer. Recently improved clinical outcomes are reported using IGBT for the advanced cervical carcinomas [13-19]. GEC-ESTRO working group recommend using MRI for determining high-risk clinical target volume (HR-CTV) and intermediate-risk CTV (IR-CTV) because MRI is superior to CT for delineating the normal anatomy of the female pelvis and for identifying cervical carcinoma extension [19-22]. However, practically majority of institutions do not have access to an MRI unit every time of brachytherapy treatment. In many circumstances CT scanners are often more widely available than MRI, therefore Viswanathan et al. developed guidelines for standard contouring of HR-CTV based on CT images [23]. From 2008 we introduced CT imaging in gynecological brachytherapy but continued to use Manchester system for dose calculation. In this study, we analyzed correlations between clinical outcome and dose of HR-CTV contoured based on CT images.

## Methods

Patients included in current study are females with cervical carcinoma treated by primary radiation therapy including brachytherapy with or without concurrent chemotherapy from April 2008 to December 2010. As mentioned above, our department introduced CT imaging in the process of high-dose rate intracavitary brachytherapy (HDR-ICBT) for cervical cancer from 2008. Sixty two patients were identified who had CT image after insertion of brachytherapy applicator and 9 patients were excluded because of having distant disease beyond pelvis and another 2 patients were excluded because they were treated by combination of ICBT and interstitial brachytherapy (ISBT). Therefore current study consisted of 51 patients. All patients underwent pelvic examination, cystoscopy, pyeloureterography, chest X-ray/CT, pelvic CT/magnetic resonance image (MRI), and blood test. Maximum tumor diameters were measured based on the CT/MRI findings. All biopsy specimens were diagnosed in Department of Pathology of our hospital.

### Treatment

Principles of management of the cervical cancer in this institute were described elsewhere [24]. The treatment policy for locally advanced cervical cancer is concurrent chemoradiation therapy (cCRT) with chemotherapy regimen of weekly cisplatin (40 mg/m^2^/week) or cisplatin (50 mg/m^2^/3 weeks) plus oral S-1 (80–120 mg/body/day). Concurrent chemoradiotherapy was not performed in the patients with insufficient renal function (serum creatinine > 1.5 mg/dl) and aged over 75 years. Supportive treatments such as blood transfusions were encouraged during radiotherapy.

### Radiotherapy

EBRT was delivered by 3D conformal technique with linear accelerator (Clinac iX, Varian Medical System, Palo Alto, CA) using 15 MV photon beam. Treatment planning was based on CT images of 3 mm slice thickness taken by Aquilion LB CT scanner (Toshiba Medical Systems, Japan). The common EBRT portals included whole uterus, as well as parametrium, upper part of vagina down to the level of lower border of obturator foramens, and the draining pelvic lymph nodes up to the level of the common iliac (L4/5 junction). The nodal CTV included internal (obturator and hypogastric), external, and common iliac lymph nodes as well as presacral lymph nodes down to the level of S3. If the primary lesion involved lower third of vagina or there were clinically palpable metastatic inguinal nodes, inguinal regions were also included in EBRT fields. The initial 20–40 Gy was delivered to the whole pelvis with a 4-field box and then pelvic irradiation with a 4 cm-width of central shield (CS) being ensued reducing organ at risk (OAR) exposure. The initiation of CS was depend upon tumor shrinkage. Every week tumor response was accessed by attending radiation oncologist by physical examination. For early responding tumor width of which was smaller than 4 cm after having received 20 Gy of EBRT, CS was initiated. For late responding tumor width of which was larger than 4 cm at 20 Gy, EBRT was continued until tumor width became smaller than 4 cm. For tumors in which response of radiation was unfavorable, CS did not introduced. Total pelvic side wall dose was 50 Gy in 25 fractions. After the CS was inserted, HDR-ICBT was performed in 1–2 sessions/week, but EBRT and HDR-ICBT were not carried out on the same day. All brachytherapy was carried out by ^192^Ir remote after loading system (RALS, MicroSelectron HDR™, Nucletron, Veennendaal, The Netherlands). ICBT with tandem + ovoid applicators without shielding was performed with a prescribed dose of 6 Gy in point A using Manchester method. A tandem-cylinder was used in the cases with a vaginal involvement exceeding more than one-third of total vaginal length. At each brachytherapy session, CT image of 3 mm slice thickness was taken by a large bore CT simulator (Aquilion™, Toshiba, Tokyo, Japan) situated in operating room with the patient lying in lithotomy position with the applicators in place. Before the acquisition of CT, bladder was filled with 100 ml of saline. Emptiness of rectum was checked at the time of gynecological examination before insertion of the applicators. For dose calculation of ICBT, Oncentra^®^ (Nucletron, Veennendaal, The Netherlands) was used. HR-CTV was determined based on CT images according to Viswanathan’s contouring guidelines [23]. Rectum and bladder were contoured as OARs. Dose constraints for OARs were determined as followed; D_2cc_ bladder < 90 Gy EQD_2_, D_2cc_ rectum < 75 Gy EQD_2_. In order to fulfill these dose constraints for OARs, tumors with insufficient response after EBRT and required 50 Gy of EBRT without CS generally could only afford two times of brachytherapy sessions while tumors with sufficient response and started CS only after 20 Gy of EBRT could undergo four or even five times of brachytherapy sessions. The workload with using CT-based IGBT required only additional several minutes for contouring targets and OARs compared with conventional X-ray based 2D planning.

### Follow-up

All patients were evaluated weekly for toxicity during radiotherapy through physical examination and blood tests. CT and/or MRI scans and cytology were performed 1–3 months after radiotherapy, and physical examination and blood tests were performed regularly every 1–6 months.

### Statistical analysis

Overall survival rate was estimated from the start of radiation therapy to the date of death or of the last follow-up. Progression-free survival rate was estimated to the date of any disease relapse considered as an event. Patients without relapse who died of another disease or still alive were censored at the time of death or last follow-up. Local control rate which includes central and parametrium relapses was considered as an event, and censored at the time of death, non-local relapse, or last follow-up. Overall survival, Progression-free survival, and local control rate were calculated by the Kaplan-Meier method.

For adding dose of EBRT and HDR-ICBT, the equivalent dose in 2 Gy fractions (EQD_2_) [11] according to LQ model [25] was calculated by the following formula:

$$\mathrm{EQ}D_{2}=\frac{\mathrm{Nd}\left(1+\frac{d}{\alpha /\beta }\right)}{1+\frac{2}{\alpha /\beta }}$$

The parameter *N* indicates the number of fractions and *d* the dose per fraction. For calculating tumor doses, α/β was assumed as 10 Gy. Because after insertion of CS most of the primary disease did not receive EBRT, EQD_2_ of EBRT before the initiation of CS was added to the EQD_2_ of HDR-ICBT. As calculation of HDR-ICBT was based on CT taken by each brachytherapy session, EQD_2_ at every fraction was calculated and added together.

The survival curves were compared by the log-rank test. For univariate analysis, all of the variables were dichotomized at the median. Statistical significance was set to less than 0.05 as usual. All of the statistical analyses were performed using SPSS Statistics version 18.0 (SAS Institute, Tokyo, Japan).

This retrospective study was approved by the institutional review board of the National Cancer Center.

## Results

**Figure 1:**
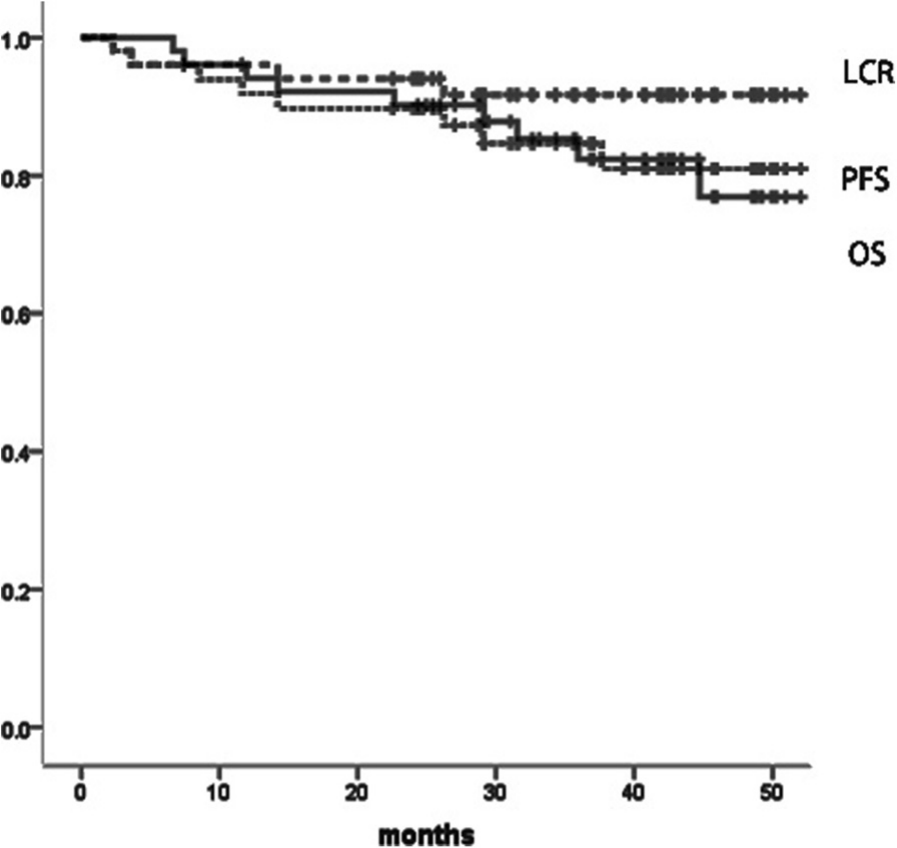
Kaplan-Meyer curves of local control rate (LCR), progression survival (PFS), and overall survival (OS).

**Figure 2:**
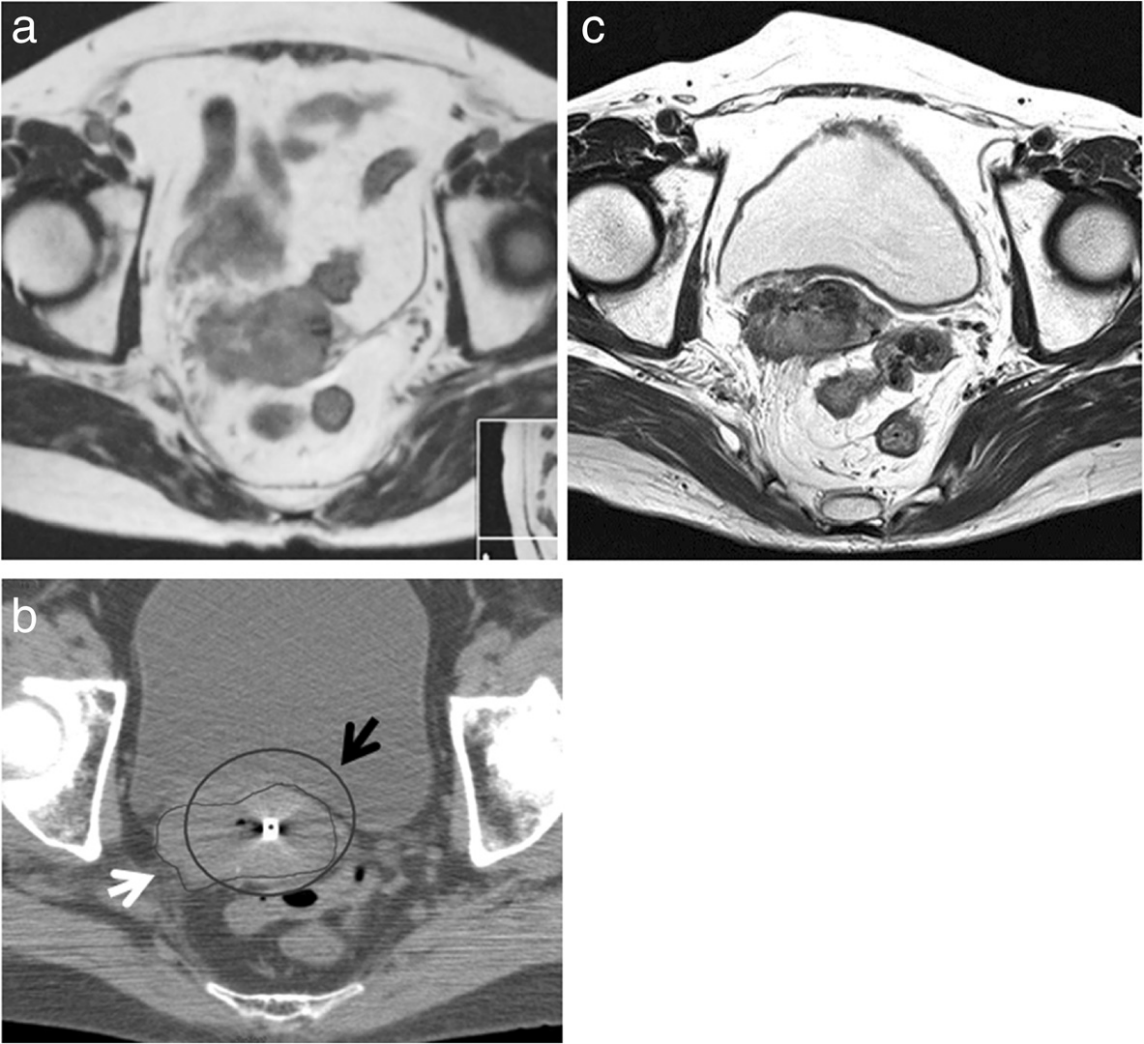
**Representative images of patient who experienced local relapse. a**. Axial MR T2 weighted image before treatment. Tumor extends to right pelvic wall. **b**. Axial CT image at the first session of intracavitary brachytherapy (ICBT). Tumor still extends to right pelvic wall after 40 Gy of whole pelvic EBRT. Black arrow represents isodose line of 60 Gy (EQD_2_) and white arrow HR-CTV at the time of brachytherapy. **c**. Axial MR T2 weighted image 3 months after completion of chemoradiotherapy. Persistent disease was found in right parametrium.

**Figure 3:**
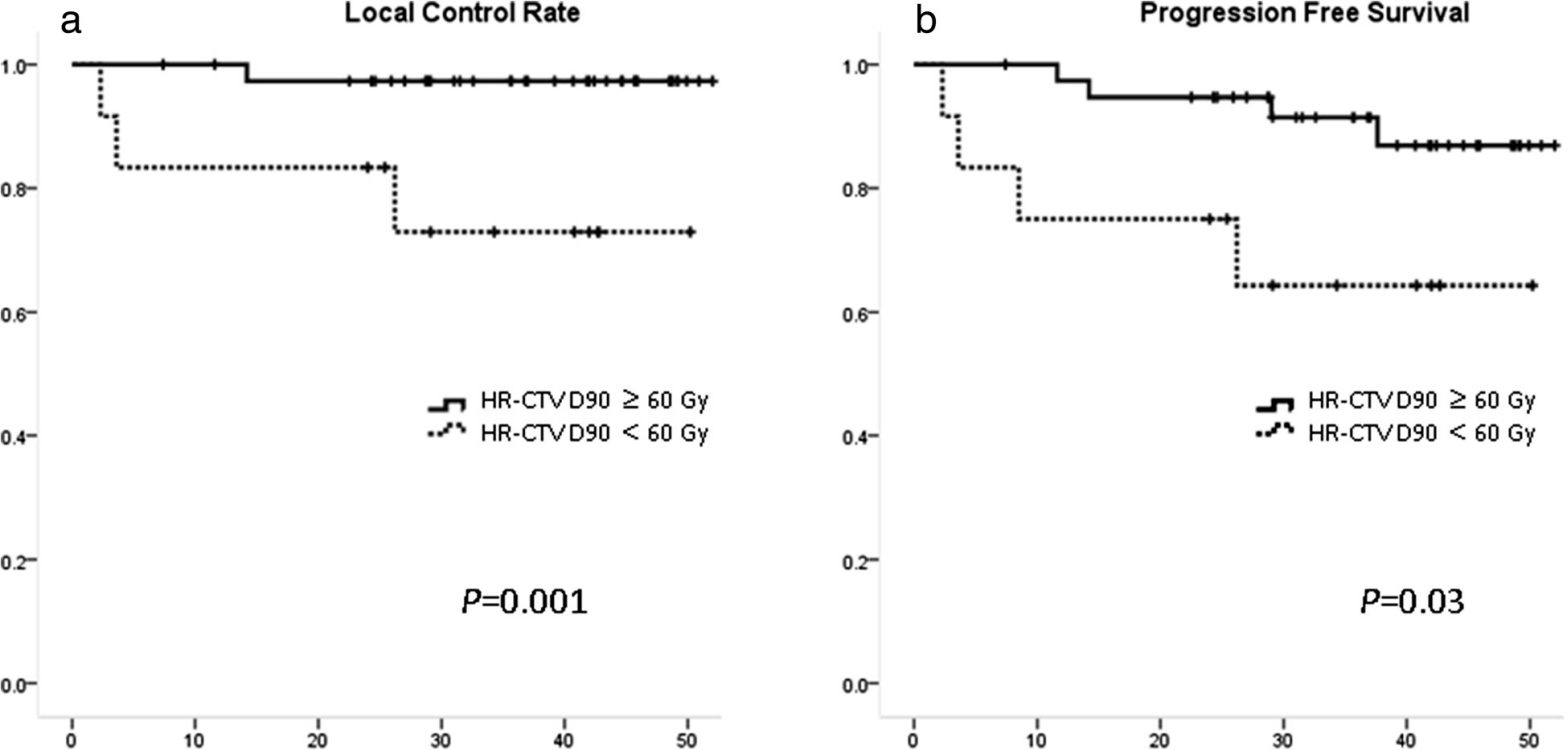
**Local control rate (LCR) and progression free survival (PFS) curve stratified by HR-CTV D90 60 Gy (EQD2). a**. Local control rate (LCR) stratified by HR-CTV D_90_ 60 Gy (EQD_2_). **b**. Progression free survival (PFS) stratified by HR-CTV D_90_ 60 Gy (EQD_2_).

Among 51 patients included in this study, 42 patients were alive at the time of the analysis and 39 were alive without disease recurrence (December 2012). The pretreatment characteristics of the 51 patients are summarized in Table 1. Treatment details were summarized in Table 2. Among 30 patients who received concurrent chemotherapy, 9 patients received cisplatin plus S-1. The median value of EQD_2_ for FIGO I/II/III/IVA was 64.55 Gy, 64.97 Gy, 64.68 Gy, and 63.35 Gy, respectively. The median follow-up length of living entire patients was 39.2 months (range, 24.3-52.0 months). Three-year Overall survival, Progression-free survival and local control rate were 82.4%, 85.3% and 91.7%, respectively (Figure 1). At the time of analysis 39 patients were alive without disease recurrence, while 5 patients died because of cancer and 4 due to other reasons without any evidence of cervical cancer. Eight out of 51 patients (15.7%) experienced persistent disease or disease recurrence after definitive radiotherapy (one, two, three, and two patients in FIGO I/II/III/IVA, respectively). Two patients recurred at only local site, 2 both local and distance simultaneously, and 4 distant only. No one experienced regional lymph node recurrence. Among seven FIGO stage IVA patients, one patient experienced local recurrence and eventually died of disease, one experienced single lung metastasis which was successfully salvaged by six cycles of carboplatin and paclitaxel followed by stereotactic radiation therapy for lung metastasis, and one elderly patient died from chronic kidney dysfunction without any evidence of disease recurrence. Figure 2 shows example of patient who experienced local recurrence. Tumor extended to pelvic wall at diagnosis (Figure 2a). The right lateral part of HR-CTV was not covered by isodose line of 60 Gy equivalent dose in 2 Gy per fraction (EQD_2_, Figure 2b). In axial MR image 3 months after completion of treatment (Figure 2c), the persistent disease was found in the area not sufficiently treated by brachytherapy. On univariate analysis, both local control rate and Progression-free survival was significantly favorable in those patients with D_90_ for HR-CTV equal to or greater than 60 Gy (Figure 3; p = 0.001 and 0.03, respectively). The number of patients with HR-CTV D_90_ < 60 Gy and ≥ 60 Gy was 12 and 39, respectively. Median volume of HR-CTV at the first application of brachytherapy in each group was 31.8 ml and 21.1 ml, respectively and patients with HR-CTV D_90_ < 60 Gy had statistically larger volume compared with that of patients with HR-CTV D_90_ ≥ 60 Gy (p = 0.022). Three-year local control rate and Progression-free survival for those whose HR-CTV D_90_ < 60 Gy was 72.9% and 64.3% whereas that of patients with HR-CTV D_90_ ≥ 60 Gy was 97.3% and 91.5%, respectively. Progression-free survival was significantly favorable in those patients when the maximum length from tandem to the margin of HR-CTV at first ICBT was shorter than 3.5 cm (p = 0.042).

**Table 1:** **Patients characteristics** (**n** = **51**)

**Characteristics**		**No. of patients**
Age	Median (range)	62 (28-90)
FIGO stage	I/II/III/IVA	10/15/19/7
Vaginal invasion	Yes	19
	No	32
Parametrium invasion	Yes	33
	No	18
Corpus invasion	Yes	15
	No	36
Pyometra	Yes	6
	No	45
Pelvic LN metastasis	Yes	11
	No	40
Pathology	Scc	48
	Adeno	3
Initial tumor size (cm)		4.5 (1.8-7.7)
Pre treatment Scc (mg/dl)		7.0 (0.9-94.2)

*LN* lymph node.

**Table 2:** Treatment details

EBRT* central pelvic dose (Gy)	Median (range)	30 (20-50)
HDR-ICBT^†^ dose for point A	Median (range)	24 (12-30)
Applicor type	Tandem + ovoid	42
	Tandem + cylinder	9
Concurrent chemotheraphy	Yes	30
	No	21
TTT^††^ (days)	Median (range)	42 (36-67)
Volume of HR-CTV at first ICBT (ml)	Median (range)	23.3 (8.3-100.8)
Maximum diameter of HR-CTV at first ICBT (mm)	Median (range)	46.9 (32.2-77.5)
Maximum length from tandem to edge of HR-CTV at first ICBT (mm)	Median (range)	29.2 (18.0-51.9)
EQD_2_^ll^ of point A	Median (range)	62 (52-72.3)
EQD_2_^ll^ of HR-CTV D_90_^**^	Media (range)	65.0 (52.7-101.7)

*EBRT: external beam radiation therapy.

^†^HDR-ICBT: high-dose rate intracavitary brachytherapy.

^††^TTT: total treatment time.

II EDQ_2_: equivalent dose in 2 Gy fractions.

**HR-CTV D_90_: dose covering 90% of the HR-CTV.

### Treatment related toxicities

One patient experienced sigmoid colon perforation 1 month after completion of radiotherapy which required colostomy. Because cumulative dose for sigmoid colon D_2cc_ was only 43.8 Gy (EQD_2_, α/β = 3 Gy) and development of the perforation was rather too early, it was implausible that radiation played a major role developing this severe morbidity. Two patients developed grade 2 proctitis and none experienced greater than grade 2 cystitis or vaginitis.

## Discussion

In the current study, definitive radiotherapy using traditional Manchester method with or without concurrent chemotherapy for cervical carcinoma resulted in favorable local control with only 4 local recurrences (7.8%).

Since the introduction of the concept of IGBT [10-12], several improved clinical results have been reported [13-18]. It is recommended in GEC-ESTRO working group that MRI should be used to determine IR-CTV and HR-CTV because of its superiority of tissue discrimination over CT image [20-22,25]. However, it is even now hard for most of brachytherapy suits to prepare MRI instruments for the use of every brachytherapy procedure for cervical cancer. As an alternative and practical solution, Viswanathan et al. proposed a guideline to contour HR-CTV based on CT images [23]. Current study was to the best of our knowledge first report which validated this CT based HR-CTV contouring guideline in clinical practice. Schmid et al. reported interesting study concerning the feasibility of transrectal ultrasonography for identifying HR-CTV in comparison with MRI [26]. However authors still believe utilizing CT for brachytherapy is the most realistic solution for the future evolution of image-guided brachytherapy for cervical cancer because of its prevalence and reproducibility.

Dimopoulos et al. analyzed the relationships between dose-volume histogram (DVH) and local control using MRI-based IGBT for cervical cancer and found out that the D_90_ for HR-CTV greater than 87 Gy resulted in excellent local control [13]. In current study, the cut-off value of D_90_ was 60 Gy and it was much lower than what Dimopoulos et al. pointed out. It has been known that Japanese centers use lower cumulative dose schedules with shorter overall treatment time (OTT) than those of US and Europe [27,28]. Recently Toita et al. showed the efficacy of Japanese schedule in a series of multicenter prospective trials in which Stage I and II with small (<4 cm) tumor diameter can be effectively treated by BED 62 Gy_10_ (JAROG0401/JROSG04-2) [29] and Stage III/IVA by BED 62–65 Gy_10_ at point A (JCOG1066) [30]. Therefore it is reasonable that in current study the cut-off value is much lower than Vienna group. In addition 60 Gy could be used as a target dose for HR-CTV D_90_ in institutions which perform IGBT with Japanese schedule. However further evidence must be accumulated in order to validate the value of HR-CTV D_90_ ≥ 60 Gy in Japanese schedule.

In current study it was revealed that PFS was significantly favorable if the maximum length from tandem to the margin of HR-CTV at the first ICBT was shorter than 3.5 cm. Therefore if the maximum distance between uterine cavity and margin of HR-CTV is longer than 3.5 cm at the first session of brachytherapy, application of image-guided brachytherapy or combined intracavitary/interstitial brachytherapy [16,31-33] would improve clinical results.

From current study, it was demonstrated that favorable local control could be achieved for tumors with HR-CTV D_90_ ≥ 60 Gy using conventional Manchester method. However for tumors with delayed response after EBRT and HR-CTV D_90_ could only be under 60 Gy by Manchester method, further treatment improvement is warranted. In this context, maximum length from tandem to the rim of HR-CTV ≥ 3.5 cm could be used as a cut-off point where ISBT would play an important role. Currently in our institution tumors of which maximum length from tandem to the rim of HR-CTV is longer than 3.5 cm at the time of brachytherapy are treated by the combination of ICBT and ISBT or ISBT alone. Improvement of clinical results after the introduction of the combination of ICBT and ISBT compared with conventional technique will be reported elsewhere.

This study has several limitations. This is a result from single retrospective study with a limited follow-up period and HR-CTV was determined based on CT images rather than MR images. Viswanathan et al. compared CT based and MRI based CTV and concluded that the width of CT based CTV was larger than that of MRI [23]. Therefore HR-CTV contoured based on CT in this study may overestimate the tumor volume in lateral direction. This may be part of the reason of lower cut-off value of HR-CTV D_90_ in this study. However it will take long before MRI will be available in majority of brachytherapy suit. At present as current standard for IGBT is based on MRI, IGBT is not so popular after its introduction in the treatment of cervical cancer brachytherapy because MRI itself is not prevalent yet. Therefore it is worth accumulating evidence that IGBT based on CT image could also achieve favorable clinical results if used properly.

## Conclusions

Dose-volume relationship was found in CT-based intracavitary brachytherapy for cervical carcinoma in Japanese schedule. Further improvement could be expected for cervical cancers with insufficient response after EBRT. For such tumor, ISBT would play an important role and should be investigated.

## Abbreviations

EBRT: External beam radiotherapy; HR-CTV: High-risk clinical target volume; IR-CTV: Intermediate-risk clinical target volume; HDR-ICBT: High-dose rate intracavitary brachytherapy; ISBT: Interstitial brachytherapy; cCRT: Concurrent chemoradiation therapy; CS: Central shield; OAR: Organ at risk; EQD_2_: The equivalent dose in 2 Gy fractions.

## Competing interests

The authors declare that they have no competing interests.

## Authors’ contributions

NM, AW, SN, HO, and JI have made substantial contributions to conception and design. NM and JI have been involved in drafting the manuscript or revising it critically for important intellectual content. MM, MS, KI, YI, and TK participated in acquisition and interpretation of data. All authors read and approved the final manuscript.

## Pre-publication history

The pre-publication history for this paper can be accessed here:

http://www.biomedcentral.com/1471-2407/14/447/prepub
